# Family-based association study of *DRD4* gene in methylphenidate-responded Attention Deficit/Hyperactivity Disorder

**DOI:** 10.1371/journal.pone.0173748

**Published:** 2017-03-10

**Authors:** Patrick Wing-leung Leung, Janice Ka Yan Chan, Lu Hua Chen, Chi Chiu Lee, Se Fong Hung, Ting Pong Ho, Chun Pan Tang, Robert K. Moyzis, James M. Swanson

**Affiliations:** 1 Department of Psychology, The Chinese University of Hong Kong, Hong Kong, PRC; 2 The Duchess of Kent Children’s Hospital at Sandy Bay, Hospital Authority, Hong Kong, PRC; 3 Department of Psychiatry, University of Hong Kong, Hong Kong, PRC; 4 Kwai Chung Hospital, Hospital Authority, Hong Kong, PRC; 5 Department of Psychiatry, The Chinese University of Hong Kong, Hong Kong, PRC; 6 Department of Biological Chemistry, University of California, Irvine, California, United States of America; 7 Department of Pediatrics, University of California, Irvine, California, United States of America; Georgetown University, UNITED STATES

## Abstract

The 48-basepair (48-bp) variable number tandem repeat (VNTR) polymorphism in exon 3 of the dopamine receptor D4 gene (*DRD4*) is implicated in the etiology of attention-deficit/ hyperactivity disorder (ADHD). In particular, ADHD in European-ancestry population is associated with an increased prevalence of the 7-repeat (7R) allele of the exon 3 VNTR. However, it is intriguing to note that the 7R allele has been found to be of very low prevalence in the Chinese general population. In a previous case-control study, our research team had found that the 7R allele was similarly absent in Chinese ADHD children in Hong Kong. Instead, there was an increased prevalence of the 2R allele in Chinese ADHD children. Interestingly, in Asian samples, the 2R allele had been found to be an evolutionary derivative of the 7R allele with equivalent biochemical functionality. So, the finding of an association between ADHD and 2R allele in Chinese population does not exactly contradict the original 7R allele finding in European-ancestry population. However, given the potential pitfall of population stratification in the previous case-control design, this current study tested the 2R allele and ADHD association using a methodologically more rigorous family-based approach on 33 Chinese ADHD probands who had favorable clinical responses to stimulant medication (methylphenidate). Haplotype Relative Risk (HRR) analysis and Transmission Disequilibrium Test (TDT) both showed a significant preferential transmission of the 2R allele from the biological parents to ADHD probands (*p*_one-tailed_ = 0.038, OR = 2.04; *p*_one-tailed_ = 0.048, OR = 2.29, respectively). A second hypothesis speculates that it is the deviation, including 7R and 2R alleles, from the conserved ancestral 4R allele which confers risk to ADHD. Thus, a preferential transmission of non-4R alleles, against the 4R allele, from biological parents to their ADHD probands is predicted. Both HRR analysis and TDT confirmed such prediction (*p*_one-tailed_ = 0.029, OR = 2.07; *p*_one-tailed_ = 0.032, OR = 2.43, respectively). This study re-confirmed the original finding of a previous study that in Chinese population, the 2R allele of the *DRD4* exon 3 VNTR was related to ADHD. This endorses the general thesis that *DRD4* exon 3 VNTR polymorphism is related to ADHD, despite that the exact length or number of repeats of the associated alleles varies across ethnicity. This in turn supports the dopamine dysregulation theory of ADHD.

## Introduction

Attention-deficit/hyperactivity disorder (ADHD), a neuropsychiatric disorder, is characterized by age-inappropriate inattention, hyperactivity and impulsivity, with male to female ratio ranging from 3:1 to 9:1 [1]. It is among the most prevalent mental health problems of children with a worldwide prevalence of about 5% [2]. Previously, ADHD was once thought to be a Western condition, encouraged by the permissiveness of the Western culture [3]. This previous view implies that ADHD may be absent in Chinese culture, in which there is more emphasis on orderly and placid behaviour. However, studies find that ADHD is no less prevalent in Chinese population with prevalence estimates of around 4%—6% in Chinese communities of Hong Kong and Taiwan [4,5]. Currently, ADHD is considered as a complex, multifactorial disorder with multiple biological etiologies, including genetics. The latter’s involvement is confirmed by various family, twin and adoption studies, with heritability estimate being 76% in one meta-analysis [6]. The biological etiologies, including genetics, underline the universality of this disorder, ADHD.

Despite the high estimated heritability of ADHD, genome-wide association studies (GWAS) have so far failed to detect any consistent polymorphism related to ADHD at the genome-wide significant level [7–12]. Experiences with the more successful GWAS studies involving such psychiatric and medical disorders as bipolar disorder and diabetes suggest a required sample size of over 4,000 to implicate a few loci and up to 60,000 to detect a larger set of genes [13,14]. Such sample sizes do not seem to be achievable for ADHD studies in the near future. Multi-centre collaboration may be required. However, given the modest inter-rater reliability in psychiatric diagnosis, variability in diagnostic practice and referral bias across multiple sites, multi-centre collaboration will lead to additional phenotypic (and possibly genotypic) heterogeneity between datasets, resulting in increased “noise” and reduced statistical power [15].

Instead, the traditional candidate gene approach, which is theory-driven and requires much smaller sample sizes to achieve adequate statistical power, has proposed genes associated with ADHD, based upon the dopamine (DA) dysregulation theory [16]. The stimulant medication (e.g., by methylphenidate), effectively treating ADHD symptomatically, binds to the dopamine transporters in the presynaptic membrane and blocks the transporter’s ability to clear DA from the synaptic space. This “mechanism of action” of the stimulant medication implicates that DA as a neurotransmitter may be involved in the etiology of ADHD. DA neurons transmit DA from mid-brain regions to the other parts of the brain via three major pathways, i.e., the nigrostriatal, mesocorticolimbic and tuberoinfundibular pathways. DA within these pathways regulates functionally and structurally various cortical and basal ganglia loops, the disruption of which produces attentional and motivational difficulties characteristics of ADHD. Animal models of ADHD also show dysregulation of DA functions. The behaviour of “ADHD” mice is normalized upon administration of stimulant medication. Brain imaging studies similarly suggest altered regulation of striatal DA levels. Two neuroimaging studies with Chinese ADHD children have identified frontal cortical regions, which are rich in dopamine, as sites related to ADHD [17,18].

Among the dopamine system genes, dopamine receptor D4 gene (*DRD4*) is the most extensively investigated with three quarters of its studies producing consistently positive results [19]. Dopamine receptor D4 is a G protein-coupled receptor and belongs to the dopamine receptors family (D1-D5). Interestingly, it is expressed in the brain prefrontal cortex, including anterior cingulate and orbitofrontal cortex, which involves regions also predominantly affected in ADHD [20,21]. The *DRD4* carries a polymorphism, which is a 48-basepair (48-bp) variable number tandem repeat (VNTR) and is located at the exon 3 of the gene. The alleles of this polymorphism range from 2 to 11 repeats with the 4-repeat (4R) allele being the most prevalent [22,23]. Following the first association study between ADHD and *DRD4* exon 3 7R allele published in 1996 [24], several subsequent meta-analyses have consistently confirmed an increase of the 7R allele in ADHD probands, based upon both case-control and family studies in European-ancestry population [6,25,26]. Functionally, the 7R allele, compared to the more common 4R allele, exhibits a blunted ability to reduce cyclic adenosine monophosphate (cAMP) level, thus requiring higher dopamine concentration for comparable reduction [27,28]. In other words, the D4 receptor produced by the 7R allele is less efficient in neurotransmission compared to that produced by the more common 4R allele.

The population prevalence of the *DRD4* 7R allele varies considerably across ethnicity and is very low in Asians, including Chinese [22]. A series of five studies in the Chinese communities of Hong Kong, Mainland China and Taiwan with Chinese ADHD children also found similarly low prevalence of the 7R allele, if not absent [29–33]. So, an association between ADHD and the 7R allele, reported in European-ancestry ADHD probands, could not be established in their Chinese counterparts. In fact, the majority of the Chinese studies did not find any association between ADHD and any single *DRD4* VNTR allele. Qian et al. only found an association with ADHD after grouping the alleles into two separate groups (i.e., 2R to 3R as short alleles and 4R to 6R as long alleles) [33]. However, there is little justification provided to such grouping. It is not sure whether it is theoretically based or data-driven. One noteworthy exception was a case-control study by Leung et al. which uncovered a significantly increased prevalence of the 2R allele in their Chinese ADHD probands compared to controls [31]. Table 1 summarizes the major findings of these five studies with Chinese ADHD samples on *DRD4* exon 3 VNTR alleles.

**Table 1:** Summary results of genetic studies with Chinese ADHD samples on DRD4 exon 3 VNTR alleles.

	Study site	Samples	Genotyping	Results
**Qian et al (2004)**	Beijing, China	Family-based (N = 202 ADHD family trios) Case-control (N = 340 ADHD cases & 226 controls)	PCR amplification followed by running on agarose gel	7R allele absent in ADHD & control children. No significant difference in frequencies of the 2R to 6R alleles individually between ADHD & control children. Only grouping alleles into short (2-3R) and long (4-6R) alleles produced some significant differences in frequencies between probands & controls. Gender a significant moderator. Family tests failed to identify preferential transmission of any alleles.
**Leung et al (2005)**	Hong Kong, China	Case-control (N = 32 ADHD cases & 247 controls) One additional inclusion criterion: response to methylphenidate	PCR amplification followed by DNA sequencing	7R allele absent in ADHD & control children. Increase of 2R allele in ADHD children compared to controls: 33% vs 20%, X^2^(1d.f.) = 5.90, p = 0.015.
**Brookes et al (2005)**	Taipei, Taiwan	Family-based (N = 216 ADHD family trios)	PCR amplification followed by running on agarose gel	7R allele absent in ADHD & control children. TDT results indicated no significant preferential transmission of any alleles.
**Cheuk et al (2006)**	Hong Kong, China	Family-based (N = 64 ADHD family trios) Case-control (N = 64 ADHD cases & 64 controls)	PCR amplification followed by running on agarose gel	Only 1 count of 7R allele from 64 ADHD probands; absent in controls. Family-based analysis reported no significant preferential transmission of any alleles. Case-control analysis found no significant difference in allelic frequency between probands & controls.
**Qian et al (2007)**	Beijing, China	Case-control (N = 307 ADHD cases & 165 controls)	PCR amplification followed by running on agarose gel	7R allele absent in ADHD & control children. No significant difference in individual allele or genotype frequencies between probands & controls. Only grouping alleles into short (2-3R) and long (4-6R) alleles produced some significant differences in frequencies between probands & controls. Gender a significant moderator.

Interestingly, one study by Wang et al. found that when the sequences of the individual motifs of *DRD4* exon 3 alleles and their linkage disequilibrium with adjacent polymorphisms were examined, the haplotypes of the particular 2R allele in Asians were found to originate from recombination between a 4R allele and a 7R allele [34]. The D4 receptors produced by the 2R and 7R alleles also functioned similarly, displaying a likewise blunted ability to reduce cAMP level [27]. Thus, the observed increased prevalence of the 2R allele in Chinese ADHD probands is not inconsistent with the 7R allele hypothesis of ADHD in European-ancestry children, given the Asian 2R allele as a derivative from the 7R allele and their equivalence in biochemical functionality. This 2R allele/ADHD association in Chinese is an original finding new to the literature and is not shared by the few other Chinese studies reported so far (see Table 1). Nonetheless, this new finding is not totally without some indirect support. A subsequent Asian study with Korean normal children found that the 2R allele and/or 7R allele were associated with novelty seeking, a temperament trait known to be associated with ADHD [35]. Unfortunately, ADHD was not directly studied in this Korean study.

To further clarify the role of *DRD4* exon 3 VNTR polymorphism, particularly the 2R allele, in Chinese ADHD children and to overcome the limitation of population stratification of the previous case-control study by Leung et al. [31], a family-based study is conducted. Two hypotheses are proposed. First, based upon the prior finding of an increased prevalence of the 2R allele in Chinese ADHD children, we hypothesize that the 2R allele, against non-2R alleles, will exhibit a preferential transmission from biological parents to their ADHD children, as in the case of the 7R allele, against non-7R alleles, in European-ancestry ADHD children. Second, the common 4R (1-2-3-4) haplotype has been identified as the conserved ancestral allele [34,36]. Any variation from it may potentially alter biochemistry and phenotype [34,37]. It is thus further hypothesized that ADHD, instead of a specific association with an increased prevalence of the 7R or 2R allele, may be associated with any increased allelic variant that differs from the ancestral 4R/4R genotype. So, our second hypothesis predicts a preferential transmission of non-4R alleles, against the 4R allele, from parents to their ADHD children.

## Materials and methods

### Participants

Thirty-three Chinese ADHD probands, aged between 6 to 15 years (mean = 9.2, SD = 1.9), and their biological fathers and mothers participated in this study. They were recruited from child psychiatric clinics in Hong Kong. Due to the gender ratio of ADHD children in local child psychiatry clinics being around 10 (male) to 1 (female), only boys were recruited for the present study. This was because a mere few ADHD girls among a majority of boys would complicate the analysis and interpretation of the results.

ADHD was first diagnosed by experienced child psychiatrists according to the Diagnostic and Statistical Manual of Mental Disorders– 4^th^ Edition (DSM-IV). This was then followed by a structured diagnostic interview using the Parent-informant version of Diagnostic Interview Schedule of Children-4th Edition (P-DISC-4) for a broad range of childhood psychiatric disorders, including ADHD [38]. The clinical diagnosis of ADHD was confirmed by DISC-IV with 15 children (45%) meeting the criteria for the combined type, 10 (30%) for the inattentive type, and 8 (24%) for the hyperactive-impulsive type. Twenty-five of the ADHD probands (75%) had at least one or more psychiatric comorbidities, including 22 cases of externalizing disorders (oppositional defiant disorder & conduct disorder), and 29 cases of internalizing disorders (specific phobias, social phobia, generalized anxiety disorder, separation anxiety disorder, agoraphobia, obsessive-compulsive disorder & dysthymic disorder). A particular inclusion criterion was a favorable clinical response to methylphenidate for at least 3 months, as judged by the attending child psychiatrists. The Verbal IQ (VIQ) of the ADHD probands was assessed by the Hong Kong Wechsler Intelligence Scale for Children. A very conservative cutoff (VIQ below 80) was adopted for excluding children with potential mental retardation. Other exclusion criteria included autism or physical disabilities, the judgement of which was also based upon the child psychiatrists’ clinical diagnosis in their routine practice. Informed written consent was obtained from the parents for their own participation and for their children. Research ethics of this study was approved by the Joint CUHK (Chinese University of Hong Kong)—NTEC (New Territories East Cluster) Clinical Ethics Committee and the KWC (Kowloon West Cluster) Clinical Ethics Committee in Hong Kong.

### Blood collection and DNA sequencing

Venous blood was taken from each ADHD boy and his two biological parents. DNA extraction and genotyping of the *DRD4* exon 3 VNTR polymorphism were performed according to a previously described method [37,39]. For each PCR amplification reaction, 25μl PCR solution, including 100ng genomic DNA, 0.5μmol pairwise primers, 200μM dXTPs, 1 X PCR buffer (Qiagen, Valencia, CA), 1 X Q-solution (Qiagen, Valencia, CA) and 0.625U Taq DNAPolymerase (Qiagen, Valencia, CA), was simultaneously running under the following conditions: 96°C for 20 seconds, 40 cycles of 95°C for 20 seconds, 68°Cfor 1 minutes, and a final step of 72°C for 4 minutes. After eliminating excess PCR primers, the final products were directly analyzed using a standard cycle sequencing technique by ABI 3100/ABI 3700 automated fluorescence sequencer (Applied Biosystems, Foster City, CA) to obtain the genotyping information.

### Statistical analysis

The Haplotype Relative Risk (HRR) analysis [40] and Transmission Disequilibrium Test (TDT) [41] were used to test the family-based transmission of alleles from the biological parents to the ADHD probands.

## Results

*DRD4* exon 3 VNTR allele frequencies of the ADHD probands and their two biological parents are shown in Table 2. Basically, the allele frequencies of this Chinese sample of parents-child trios were dominated by two alleles, i.e., the 2R and 4R alleles.

**Table 2:** DRD4 exon 3 VNTR allele frequency of Chinese ADHD parents-child trios.

	2R	3R	4R	5R	6R
**Probands (n = 33)**	22 (33%)	2 (3%)	41 (62%)	1 (2%)	0 (0%)
**Parents (n = 66)**	35 (27%)	2 (2%)	92 (70%)	2 (2%)	1 (1%)

HRR analysis confirmed our first hypothesis, demonstrating a significant preferential transmission of the 2R allele, compared to other non-2R alleles, from the biological parents to the ADHD probands, 63% versus 45% with an odd ratio (OR) = 2.04 (χ^2^ (1, N = 132) = 3.15, *p*_one-tailed_ = 0.038,) (Table 3). TDT further confirmed this preferential transmission of the 2R allele. Twenty-three informative parental meioses were identified, in which 16 2R alleles (70%) were transmitted, compared to 7 (30%) that were not transmitted (McNemar’s χ^2^ (1, N = 66) = 2.78, *p*_one-tailed_ = 0.048, OR = 2.29) (Table 4).

**Table 3:** HRR analysis of DRD4 2R and non-2R alleles in Chinese ADHD parents-child trios.

	2R	Non-2R	Total
**Transmitted**	22 (63%)	44 (45%)	66
**Non-Transmitted**	13 (37%)	53 (55%)	66
	35	97	132

χ^2^ (1, N = 132) = 3.15, *p*_one-tailed_ = 0.038, OR = 2.04

**Table 4:** TDT of DRD4 2R and non-2R alleles in Chinese ADHD parents-child trios.

		Non-Transmitted		Total
		2R	Non-2R	
**Transmitted**	**2R**	6	16	22
	**Non-2R**	7	37	44
		13	53	66

McNemar’s χ^2^(1, N = 66) = 2.78, *p*_one-tailed_ = 0.048, OR = 2.29

Our second hypothesis predicted a preferential transmission of non-4R alleles, against the 4R allele, from biological parents to their ADHD probands. Tables 5 and 6 reported very similar results as those of Tables 3 and 4. In brief, both HRR analysis and TDT confirmed our second hypothesis of a significant preferential transmission of non-4R alleles (χ^2^ (1, N = 132) = 3.59, *p*_one-tailed_ = 0.029, OR = 2.07; McNemar’s χ^2^ (1, N = 66) = 3.38, *p*_one-tailed_ = 0.032, OR = 2.43, respectively). In the former analysis, 63% of the non-4R alleles, compared to 45% of the 4R allele, was preferentially transmitted from the parents to the ADHD children, while in the latter, 24 informative parental meioses were identified, in which 17 non-4R alleles (71%) were transmitted, compared to 7 (29%) that were not transmitted.

**Table 5:** HRR analysis of DRD4 non-4R and 4R alleles in Chinese ADHD parents-child trios.

	Non-4R	4R	Total
**Transmitted**	25 (63%)	41 (45%)	66
**Non-Transmitted**	15 (37%)	51 (55%)	66
	40	92	132

χ^2^ (1, N = 132) = 3.59, *p*_one-tailed_ = 0.029, OR = 2.07

**Table 6:** TDT analysis of DRD4 non-4R and 4R alleles in Chinese ADHD parents-child trios.

		Non-Transmitted		Total
		Non-4R	4R	
**Transmitted**	**Non-4R**	8	17	25
	**4R**	7	34	41
		15	51	66

McNemar’s χ^2^ (1, N = 66) = 3.38, *p*_one-tailed_ = 0.032, OR = 2.43

## Discussion

In a previous case-control study, the 2R allele of the *DRD4* exon 3 VNTR had been found to be of increased prevalence in Chinese ADHD children [31]. Unfortunately, the case-control design suffers from the challenge of population stratification. A methodologically more rigorous family-based study is thus preferred and conducted here. Both HHR analysis and TDT confirm our first hypothesis that the 2R allele is preferentially transmitted to Chinese ADHD children from their biological parents.

This finding of an association between ADHD and the 2R allele is superficially in discrepancy with findings from European-ancestry ADHD samples, which indicate an association with the 7R allele instead. However, the 2R allele may be as noteworthy as the 7R allele in its association with ADHD due to the evolutionary connection between the two alleles in Asians and their similar biochemical functionality. Therefore, our current finding of an association between ADHD and the 2R allele, based on a methodologically more rigorous family-based design, reinforces that of the earlier case-control study [31] and additionally strengthens the original 7R allele hypothesis of ADHD developed from European-ancestry samples. This in turn supports the dopamine dysregulation theory of ADHD, which hints at the involvement of dopamine system genes in the etiology of ADHD, including *DRD4*.

Compared to the modest effect sizes of the 7R allele in ADHD with ORs of 1.27–1.34 estimated from meta-analysis of both case-control and family-based studies [25,26], or with ORs of 1.16–1.40 estimated from meta-analysis of family-based studies only [6], the current effect sizes of the 2R allele in our Chinese family-based study are favourable with ORs of 2.04 from HRR analysis and 2.29 from TDT.

As noted above, there are a few other Chinese studies with negative results regarding the association of ADHD and *DRD4* exon 3 VNTR individual alleles (see Table 1). Inconsistent findings are not uncommon in genetic research and the reasons can be manifold. First, there are differences in sample characteristics. The samples of the previous Leung et al.’s study and this study had one specific inclusion criterion not shared by other Chinese studies [29,30,32], i.e., persistent favorable response to methylphenidate medication (>3 months). Previous research did find the association between ADHD and dopamine genes to be more readily identifiable when non-responders to methylphenidate were excluded [42]. The choice of this refined ADHD phenotype of persistent medication responders echoes the central role of the DA dysregulation theory played in ADHD pathophysiology and etiology, i.e., the beneficial effects of stimulant treatment implicating DA dysregulation in ADHD and thus the involvement of dopamine system genes, including *DRD4*. Second, only boys are recruited in previous Leung et al.’s study [31] and this study. However, studies by Cheuk et al. and Qian et al. included both boys and girls[30,32], but gender dimorphism had been reported as the frequencies of long alleles (4-6R) and short alleles (2-3R) differed in an opposite direction by gender [30,32]. Third, the allele frequencies of the ancestral 4R allele were very high in other Chinese studies with negative findings [29,30,32] compared to those in previous Leung et al.’s study [31] and this study (75–84% vs 62–63%). In these two studies by Leung and his associates, sequencing analysis, “a gold standard”, is used to obtain genotyping information of the *DRD4* exon 3 VNTR, instead of using visualized gels to identify the allele length of the polymorphism as in other Chinese ADHD studies [29,30,32]. It is speculated that these studies may have set PCR amplification conditions (i.e., concentration of 7-Deaza-dGTP or temperature) to maximize the detection of "long" alleles compared to "short" alleles of the hard-to-amplify 48-bp VNTR of the *DRD4* gene because of the original 7R allele hypothesis. Kaiser et al. had shown that this could create a differentially bias toward "long" alleles and might "…give reproducibly wrong results in heterozygous subjects due to selective amplification of only one of the alleles" [43]. Thus, the participants’ characteristics and the genotyping methods, which are notoriously difficult and finicky, may have differed in fundamental ways across these various Chinese studies and these in turn may explain the discrepant results.

The second hypothesis of this study suggests that it is not exclusively the 7R or 2R allele that is associated with ADHD. Instead, it is any allelic variant that differs from the conserved ancestral 4R/4R genotype. So, our second hypothesis predicts a preferential transmission of non-4R alleles, against the 4R allele, from biological parents to their ADHD children. This hypothesis is confirmed by HRR analysis and TDT. However, Table 1 shows that alleles other than the 2R and 4R alleles (i.e., 3R and 5R) account for no more than 5% of the total number of alleles in either the ADHD probands or the parents. Thus, only very few 3R and 5R alleles are grouped with the 2R allele to form the non-4R alleles to test the second hypothesis. Consequently, the results of the analysis resemble very much to those testing the first hypothesis, i.e., the 2R allele versus the 4R allele. It is not sure whether if we have a much larger sample than 33 ADHD trios, then there may be a much wider spread of different alleles. Or, the *DRD4* exon 3 VNTR polymorphism of the Chinese ADHD probands and their parents recruited in Hong Kong continues to remain essentially a two-allele system of 2R and 4R alleles as in the case of this present sample. Nonetheless, this second hypothesis remains an intriguing theoretical proposition to be further empirically tested.

Our study is limited by the small sample size of 33 parents-child trios. So, our current results must be viewed cautiously, since the chance of a false positive error increases with a small sample size. Because of the small sample size, further analysis by the three ADHD types, i.e., combined, inattentive and hyperactive-impulsive, will not be meaningful. It is also not sure how our findings are generalizable to ADHD girls. As noted above, there is the possibility of a gender difference. Our current unit of analysis is with *DRD4* exon 3 48-bp VNTR. However, these repeats in fact involve different haplotypes comprising of different 48-bp motifs. Some previous studies did find some rare or novel haplotypes in the 2R, 3R, 5R, 7R and 8R alleles in ADHD children [36,37]. The exact etiological role of these rare/novel haplotypes to ADHD is still unclear. Some recent studies also look at single nucleotide polymorphisms (SNPs). One study reported that -376 C/T SNP (rs916455) of *DRD4* was found to predict the persistence of ADHD to adulthood [44]. In other words, in future studies, our genetic investigation should be expanded to include rare/novel haplotypes of the exon 3 VNTR or mutant SNPs of *DRD4*. Possibly, other candidate genes than *DRD4* involved in the dopaminergic pathway should also be included in the investigation. Finally, it must be noted that a minority of ADHD children, up to 20 or 30%, respond less satisfactorily to medication by methylphenidate, implying that the DA theory of ADHD may not be applicable to them. It is unsure whether our current findings can be generalizable to them. This explains why ADHD is considered such a complex, multifactorial disorder with multiple biological etiologies.

## Supporting information

S1 File Dataset for this study. (XLS)
